# Combinatorial effects of doxorubicin and retargeted tissue factor by intratumoral entrapment of doxorubicin and proapoptotic increase of tumor vascular infarction

**DOI:** 10.18632/oncotarget.12559

**Published:** 2016-10-11

**Authors:** Janine Stucke-Ring, Julian Ronnacker, Caroline Brand, Carsten Höltke, Christoph Schliemann, Torsten Kessler, Lars Henning Schmidt, Saliha Harrach, Verena Mantke, Heike Hintelmann, Wolfgang Hartmann, Eva Wardelmann, Georg Lenz, Bernhard Wünsch, Carsten Müller-Tidow, Rolf M. Mesters, Christian Schwöppe, Wolfgang E. Berdel

**Affiliations:** ^1^ Department of Medicine A (Hematology, Hemostaseology, Oncology and Pneumology), University Hospital of Muenster, Muenster, Germany; ^2^ Department of Clinical Radiology, University Hospital of Muenster, Muenster, Germany; ^3^ Gerhard-Domagk Institute for Pathology, University Hospital of Muenster, Muenster, Germany; ^4^ Department of Pharmaceutical Chemistry, Westfalian Wilhelms-University, Muenster, Germany; ^5^ Department of Hematology and Oncology, University Hospital Halle, Halle, Germany

**Keywords:** retargeted tissue factor, vascular targeting, vascular infarction, doxorubicin tumor entrapment

## Abstract

Truncated tissue factor (tTF), retargeted to tumor vasculature by GNGRAHA peptide (tTF-NGR), and doxorubicin have therapeutic activity against a variety of tumors. We report on combination experiments of both drugs using different schedules. We have tested fluorescence- and HPLC-based intratumoral pharmacokinetics of doxorubicin, flow cytometry for cellular phosphatidylserine (PS) expression, and tumor xenograft studies for showing *in vivo* apoptosis, proliferation decrease, and tumor shrinkage upon combination therapy with doxorubicin and induced tumor vascular infarction. tTF-NGR given before doxorubicin inhibits the uptake of the drug into human fibrosarcoma xenografts *in vivo*. Reverse sequence does not influence the uptake of doxorubicin into tumor, but significantly inhibits the late wash-out phase, thus entrapping doxorubicin in tumor tissue by vascular occlusion. Incubation of endothelial and tumor cells with doxorubicin *in vitro* increases PS concentrations in the outer layer of the cell membrane as a sign of early apoptosis. Cells expressing increased PS concentrations show comparatively higher procoagulatory efficacy on the basis of equimolar tTF-NGR present in the Factor X assay. Experiments using human M21 melanoma and HT1080 fibrosarcoma xenografts in athymic nude mice indeed show a combinatorial tumor growth inhibition applying doxorubicin and tTF-NGR in sequence over single drug treatment. Combination of cytotoxic drugs such as doxorubicin with tTF-NGR-induced tumor vessel infarction can improve pharmacodynamics of the drugs by new mechanisms, entrapping a cytotoxic molecule inside tumor tissue and reciprocally improving procoagulatory activity of tTF-NGR in the tumor vasculature via apoptosis induction in tumor endothelial and tumor cells.

## INTRODUCTION

Denekamp et al. first proposed tumor vessels and endothelial cells as a target for antitumor therapy [1]. Use of tumor vascular targeted tissue factor (TF), a central initiator of the extrinsic coagulation pathway, to induce tumor vessel infarction was proposed by Huang et al. in the Thorpe-lab [2]. Among a multitude of other target molecules on tumor vessels, Pasqualini et al. [3] revealed that small peptides containing the NGR motif (asparagine-glycine-arginine) bind to aminopeptidase N (APN; CD13). CD13 is a cell surface molecule with up-regulated expression on endothelial cells in tumors and tissues that undergo angiogenesis.

We have constructed a series of fusion proteins consisting of short NGR-peptide sequences coupled to the C-terminal end of tTF [4–8]. Among others from this series, tTF-NGR (HIS_tag_-tTF_1-218_-GNGRAHA) as a model fusion protein retains its procoagulatory activity *in vitro*, binds to the respective targets on endothelial and tumor cells, and upon intravenous infusion induces vascular infarction in blood vessels of human tumors of various histologies growing in athymic mice with subsequent tumor growth retardation or regression. Tumor vascular infarction induced by tTF-NGR can be impressively visualized by different *in vivo* or *ex vivo* imaging approaches [6, 8]. Intravenous infusion of tTF-NGR in late-stage cancer patients at dose levels without side effects was shown to reduce tumor blood flow *in situ* [5].

To further improve the therapeutic antitumor efficacy of both compounds, we started experimental combination therapy protocols of tTF-NGR with classical cytotoxic drugs such as doxorubicin. Here we report on a series of experiments examining new mechanistic aspects of combining tumor vascular infarction induced by tTF-NGR or random-PEGylated TMS(PEG)_12_ tTF-NGR and the application of doxorubicin *in vitro* and *in vivo*. Our results show that doxorubicin is effectively entrapped by tTF-NGR-induced vascular infarction inside tumor tissues leading to a longer exposure time of the tumor to the drug with increased and prolonged tumor cell apoptosis. On the other hand, doxorubicin-induced early apoptosis of tumor endothelial and tumor cells with consecutive accumulation of phosphatidylserine (PS) on the outer cell surface can increase the procoagulatory milieu for tTF-NGR as measured by a Factor X (FX) assay. Both mechanisms lead to an improved therapeutic effect of the combination over single drug treatment, completely stopping tumor xenograft growth for the duration of experimental observation, even when suboptimal doses and schedules of tTF-NGR were applied. This observation contributes to the design of randomized clinical trials with tTF-NGR in the future.

## RESULTS

### Kinetics of intra-tumoral and intra-organ doxorubicin concentrations upon different combination schedules with tTF-NGR

Doxorubicin is an anthracycline and among the most widely used antitumor cytotoxic compounds. Short intravenous infusions of doxorubicin are followed by triphasic clearance kinetics from plasma [9]. Initial distribution half-life is approx. 5–10 minutes, secondary half-life is 1–3 hours, and terminal elimination half-life can be measured at 25–50 hours. We hypothesized that tumor vessel occlusion with retargeted tissue factor tTF-NGR will entrap this organic cytotoxic molecule inside tumor tissue and thus prolong and amplify its antitumor effect.

**Figure 1:**
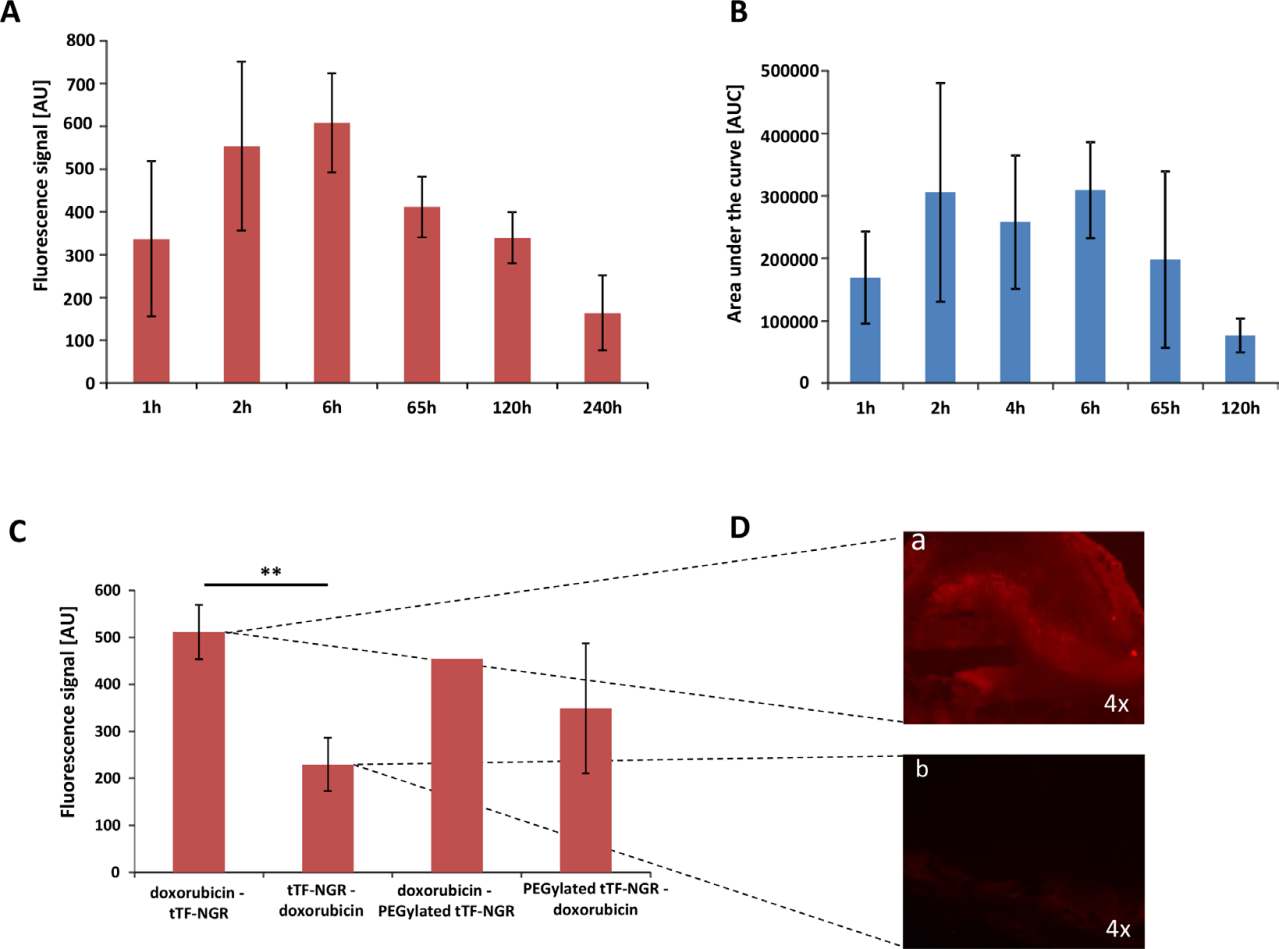
Fluorescence- and HPLC-based quantification of doxorubicin content in tumor tissue Doxorubicin was injected at a dose of 5 mg/kg bw i.v. and the intratumoral concentration was determined over a time of up to 240 hours by spectrofluorometric analysis (**A**) 1 h *n* = 3, 2 h *n* = 4, 6 h *n* = 2, 65 h *n* = 2, 120 h *n* = 5 and 240 h *n* = 2) and up to 120 hours by HPLC (**B**) 1 h *n* = 3, 2 h *n* = 2, 4 h *n* = 3, 6 h *n* = 2, 65 h *n* = 3 and 120 h *n* = 3). The maximum fluorescence signal (tissue C_max_) was obtained at 6 hours (t_max_) after injection with a following slow monophasic decrease of intratumoral doxorubicin concentrations and remaining intratumoral levels still detectable after 240 hours. tTF-NGR (1mg/kg bw i.v.) or PEGylated tTF-NGR (5 mg/kg bw i.v.) injection 4 hours before doxorubicin yielded a decreased uptake of doxorubicin into tumor tissue in comparison to a fast opposite sequence (**C**). *P*-values showed significance in uptake blockade (*p* = 0.0079, **, Mann-Whitney test). Furthermore, tumor fluorescence microscopy photographs (**D**) visualized the decrease in autofluorescence of intratumoral doxorubicin injected after tTF-NGR (D, b) over the opposite sequence injecting tTF-NGR after doxorubicin (D, a). AU, arbitrary units. All data are presented in means ± SEM.

For measuring intratumoral concentrations of doxorubicin, we have taken advantage of the autofluorescence of the molecule with excitation wavelength of 479 nm and emission wavelength of 590 nm [10]. To this end, we have used HT1080 human xenograft tumors growing subcutaneously in CD-1 athymic mice, treated the animals intravenously (i.v.) with 5 mg/kg body weight (bw) doxorubicin at an approx. tumor size of 250 mm^3^, and excised the tumors at different time points after injection. Tumor tissue was then subjected to doxorubicin concentration measurement as described in Methods. Intratumoral accumulation of doxorubicin occurred fast after injection with high fluorescence levels already measured at 1 hour after injection (Figure 1A, 1B). Intratumoral C_max_ (maximum concentration) values were obtained approx. 6 hours after injection with a subsequent slow monophasic decrease of intratumoral doxorubicin concentrations and residual tissue levels still detectable after 240 hours (Figure 1A, 1B). To establish plausibility of the model for treatment sequences, we then combined doxorubicin with tTF-NGR in a sequence applying tTF-NGR or PEGylated TMS(PEG)_12_ tTF-NGR first, subsequently followed by doxorubicin after short time intervals (4 hours interval; application sequence: tTF-NGR - doxorubicin). Experiments with this sequence showed drastically decreased uptake of doxorubicin into tumor tissue when compared with a doxorubicin - tTF-NGR (fast application: 1st doxorubicin, 2nd tTF-NGR) sequence (Figure 1C). This is easily explained by tTF-NGR-induced tumor vascular occlusion before doxorubicin is able to accumulate within the tumor. The fluorescence microscopy findings of doxorubicin - tTF-NGR versus tTF-NGR - doxorubicin sequences underline these results (Figure 1D).

**Figure 2:**
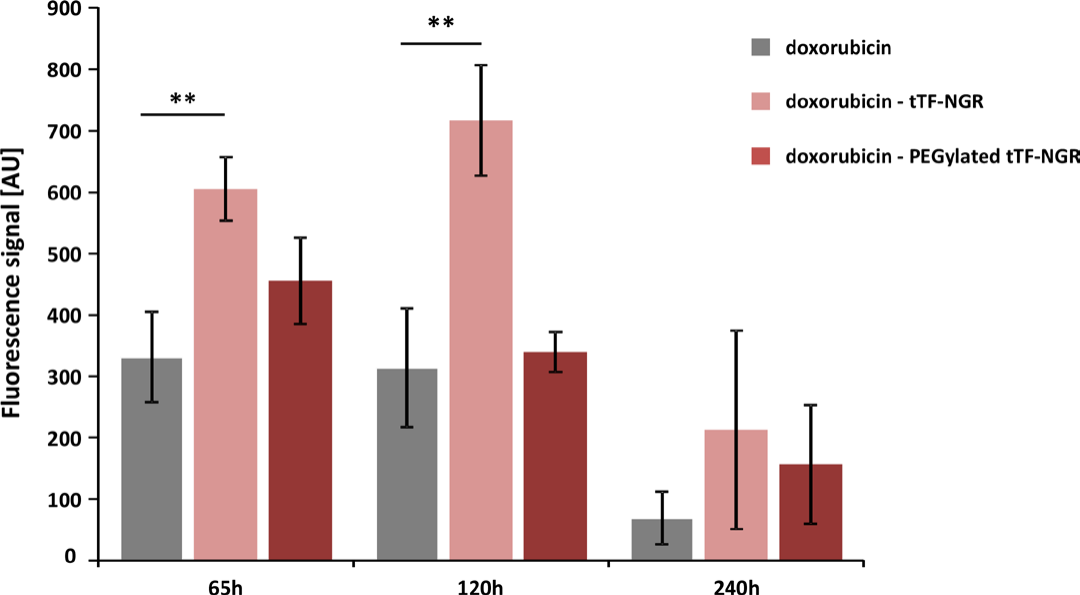
Fluorescence-based quantification of doxorubicin wash-out kinetics upon combinatorial application of doxorubicin and tTF-NGR or randomly PEGylated tTF-NGR, respectively The HT1080 tumor-bearing mice were injected with a 1st application of tTF-NGR or PEGylated tTF-NGR 6 hours after doxorubicin injection at intratumoral C_max_ of doxorubicin, and vascular occlusion was upheld by repeated application of tTF-NGR or randomly PEGylated tTF-NGR, respectively, over time. This combinatorial protocol significantly retarded wash-out times of doxorubicin from the tumor with prolonged high intratumoral drug levels in the tumor tissue after 65 and 120 hours upon doxorubicin - tTF-NGR sequences as compared to control sequences of doxorubicin - saline (*p* = 0.0043 at 65 h, *p* = 0.0095 at 120 h; **Mann-Whitney test). Non-PEGylated tTF-NGR had a more pronounced effect than randomly PEGylated tTF-NGR. Data are presented in means ± SEM. AU, arbitrary units.

However, as shown in Figure 2, injecting tTF-NGR 6 hours after doxorubicin (at the intratumoral C_max_ of the drug) and iterating vascular occlusion by repeated application of tTF-NGR over time significantly retarded wash-out times of doxorubicin from the tumor. Besides, it revealed prolonged high intratumoral drug levels in the tumor tissue when compared to control sequences of doxorubicin - saline. Non-PEGylated tTF-NGR had a more pronounced effect than random-PEGylated TMS(PEG)_12_ tTF-NGR. Thus, we could show significant intratumoral entrapment of doxorubicin by tTF-NGR-induced tumor vascular occlusion.

**Figure 3:**
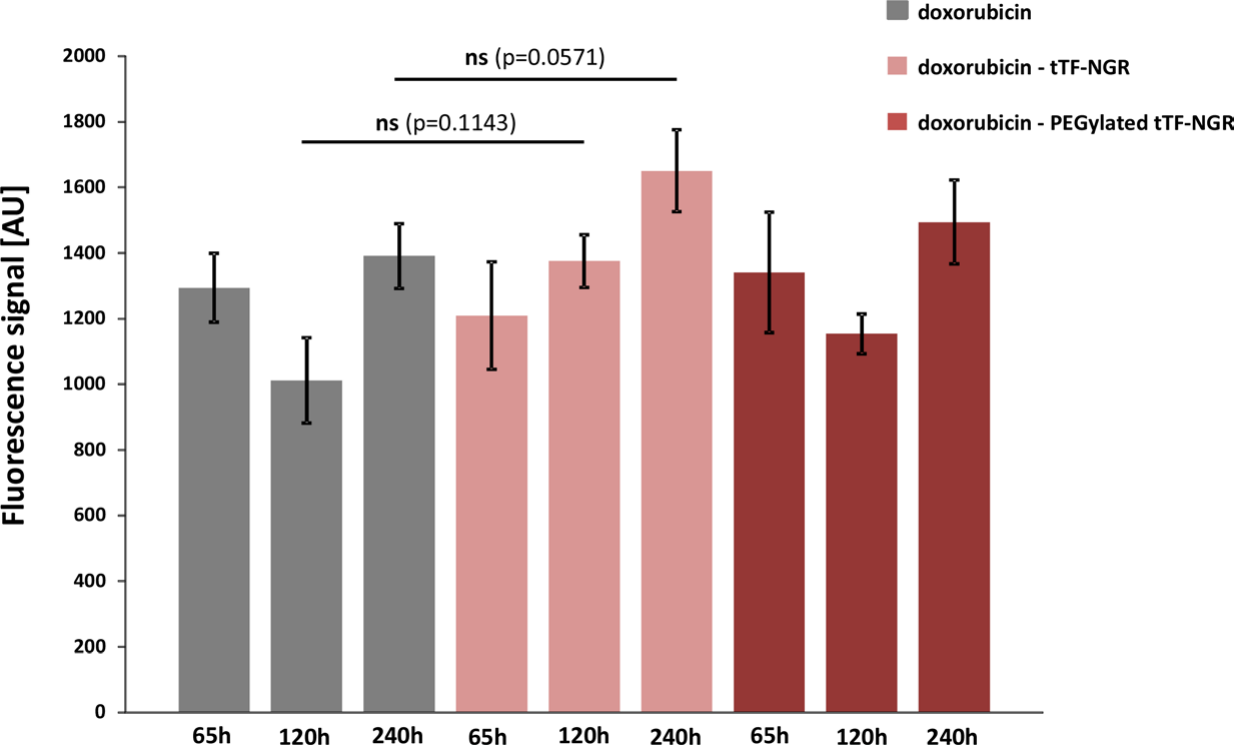
Fluorescence-based quantification of doxorubicin content in normal lung tissue For details see Figures 1 and 2. Identical time points following doxorubicin - tTF-NGR sequences were taken for comparative studies on normal organ tissues. In normal tissues such as lung no entrapment of doxorubicin by tTF-NGR could be shown, indicating that doxorubicin organ toxicity should not be altered by this combination with tTF-NGR. AU, arbitrary units. Data are shown as means ± SEM.

Identical time points following doxorubicin - tTF-NGR sequences were taken for comparative studies on normal organ tissues. In normal tissues such as lung no such entrapment of doxorubicin by tTF-NGR was observed (Figure 3), indicating that doxorubicin distribution in normal organs is not altered by this combination with tTF-NGR.

### Doxorubicin entrapped by tTF-NGR induces higher levels of tumor cell apoptosis *in situ*

**Figure 4:**
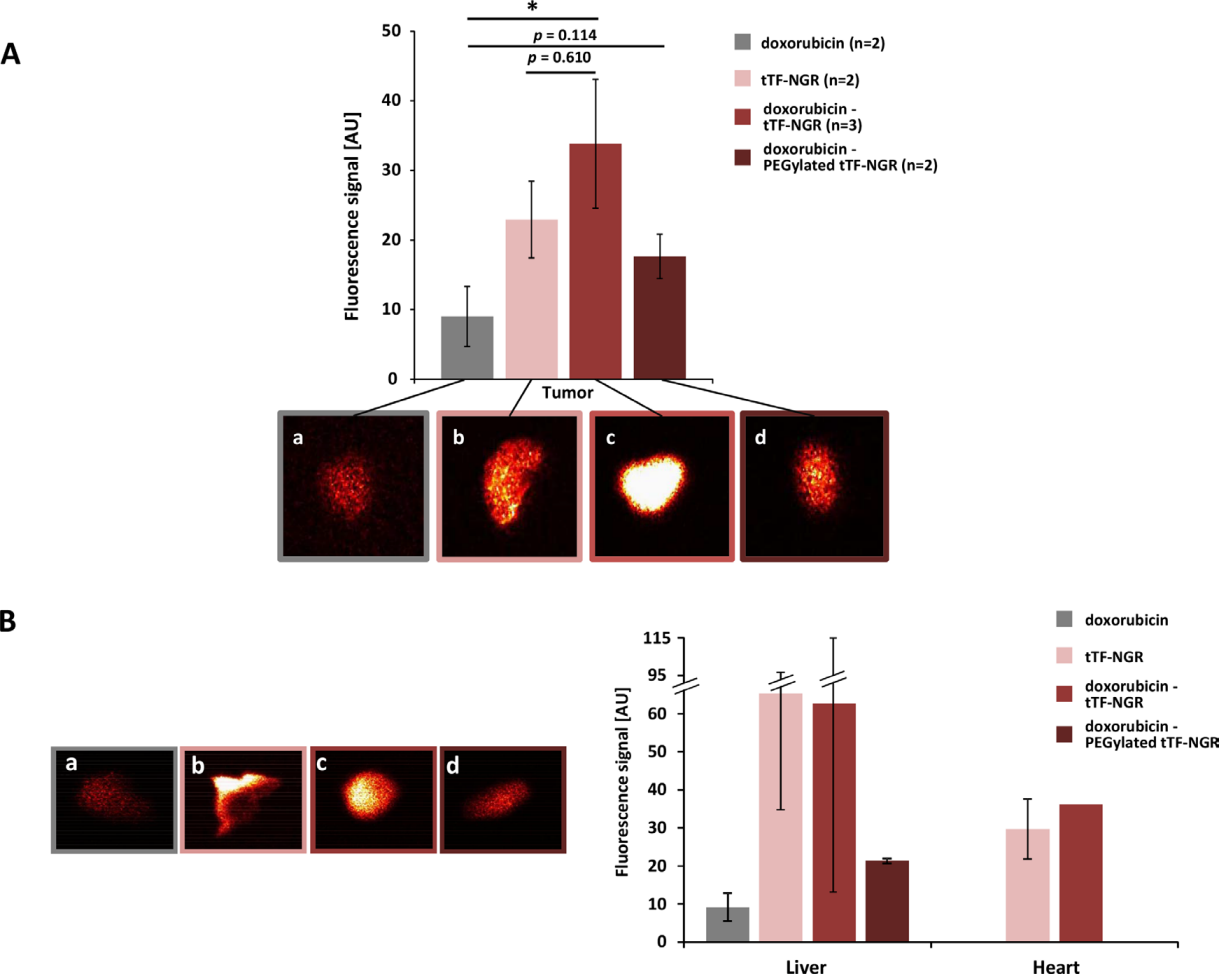
Fluorescence-based quantification of apoptosis in tumor and organ tissues *in situ* Petri dish with tumors (2–3 weeks after cell inoculation) or organs 98 hours post-injection of *Annexin-Vivo* 750 and 120 hours after start of treatment (**A**) color-coded fluorescence reflectance images: (a) doxorubicin 5 mg/kg bw (day 0); (b) tTF-NGR 1 mg/kg bw (day 0, 2, 5); (c) doxorubicin 5 mg/kg bw (day 0) followed by tTF-NGR 1 mg/kg bw (6 h after doxorubicin, and days 2, 5); (d) doxorubicin 5 mg/kg bw followed by PEGylated tTF-NGR 5 mg/kg bw (6 h after doxorubicin, and days 2, 5). High fluorescence values are observed in the tumors (A, b-d). Semi-quantitative data showed significant higher levels of tumor cell apoptosis *in situ* in mice, which received the doxorubicin - tTF-NGR than in mice receiving a doxorubicin - saline control sequence (**A**) *p* = 0.0381, *, Mann-Whitney test), whereas doxorubicin - PEGylated tTF-NGR vs. doxorubicin (*p* = 0.114) and doxorubicin vs. doxorubicin – tTF-NGR (*p* = 0.610) showed a trend in favour of the combination, but no significant tTF-NGR differences in fluorescence signal. (**B**) The liver and at lower levels the heart showed signs of *in situ* apoptosis in mice injected with tTF-NGR and doxorubicin - tTF-NGR (B, a-d, liver), but there was no quantitative difference between tTF-NGR alone or the combination of doxorubicin - tTF-NGR, respectively (B, right panel). CAVE: The absolute values cannot be compared between the single organs and tumors, since different organ sizes interfere (e.g. liver size bigger than tumor size). AU, arbitrary units. All data are presented in means ± SEM.

To test for biological consequences of this new method to accumulate doxorubicin in tumors by entrapment following vascular occlusion, we next subjected tumor tissues directly after excision to fluorescence reflectance imaging (FRI) by injecting *Annexin-Vivo* 750 as an apoptosis marker 96 h before imaging. Combinatorial treatment with the doxorubicin - tTF-NGR or - TMS(PEG)_12_ tTF-NGR sequences induced significantly higher levels of tumor cell apoptosis *in situ* than doxorubicin - saline control sequences (Figure 4A a-d). Since the liver in contrast to other normal organs also showed lower but visible signs of *in situ* apoptosis upon administration of the doxorubicin - tTF-NGR sequence (Figure 4B a-d), potential liver toxicity should be closely monitored in clinical trials applying this combination. While the heart represents the organ with dose limiting cumulative toxicity (DLT) for doxorubicin, liver represents an organ for potential DLT of tTF-NGR in the safety pharmacology series of experiments for this compound according to the ICH guidelines S6/9. Organs such as the kidneys, which are operative in metabolism or excretion of the fluorescence dye, or lungs were positive in all assays without differences (details not shown).

### Doxorubicin increases PS concentration on the outer cell surface of endothelial and tumor cells which leads to a higher procoagulatory state of the cells

After we have demonstrated a higher and longer intratumoral concentration of doxorubicin upon entrapment in tumors by tTF-NGR-induced vascular occlusion, we next performed experiments to test for reciprocal cooperative interaction of both drugs. *Ex vivo* testing had shown increased tumor cell apoptosis following doxorubicin - tTF-NGR sequence. Thus, we hypothesized that increasing phosphatidylserine (PS) externalization to the outer cell surface of endothelial and tumor cells induced by pro-apoptotic activity of doxorubicin can lead to a better procoagulatory milieu and consecutively to a more efficient FX activation by tTF-NGR. Experiments reported before have shown phospholipid/PS-dependency of the procoagulatory state [11–13], increasing PS externalization to the outer leaflet of the cellular phospholipid bilayer by pro-apoptotic maneuvers including cytotoxics [13–15], and the consequence of both, i.e. increased procoagulatory activity of endothelial cells by PS externalization [16–19]. Since in tumor vessels, the inner vascular cell layer is not only built by endothelial cells but in addition also by tumor cells in a process termed vascular mimicry [20], we have also tested the procoagulatory behaviour of tumor cells following induction of apoptosis by doxorubicin as a further mechanism explaining the combinatorial effect.

**Figure 5:**
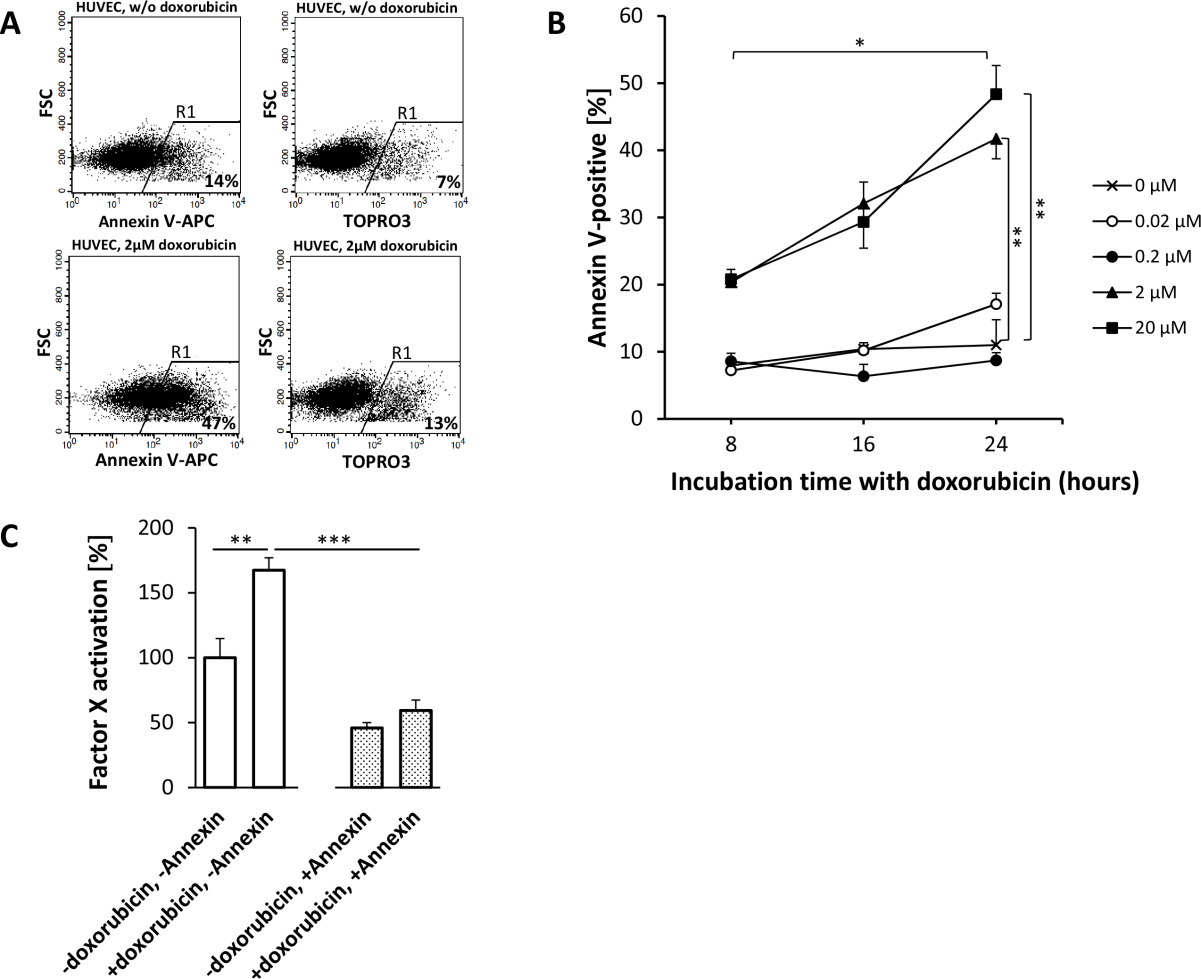
Doxorubicin-induced phosphatidylserine (PS) externalization on HUVEC enhances the procoagulatory activity of tTF-NGR APC Annexin V stains early apoptotic cells, which display PS on their surface, TOPRO3 iodide stains necrotic cells (**A** and **B**). The amount of PS on the outer surface of HUVECs correlates with the dosage of and incubation time with doxorubicin. Upon 16 hours treatment with 2 μM doxorubicin, the fraction of PS positive cells rises from 14% (non-treated cells) to 47%, whereas the number of necrotic cells only increases moderately from 7% to 13% (A). For the chart in (B), the percentage of stained cells in R1 has been reduced by the fraction of unstained cells in R1 (not shown). Doxorubicin concentrations of 2 μM and 20 μM display significant differences to controls for all incubation times (2 μM: p_8h_ = 0.0007, p_16h_ = 0.0180, p_24h_ = 0.0030 (**); 20 μM: p_8h_ = 0.0022, p_16h_ = 0.0198, p_24h_ = 0,0028 (**); two-tailed *t*-test). For these concentrations, 24 h incubation leads to significantly (*p* < 0.05, *) elevated PS exposure when compared to 8 hours. Lower concentrations do not show efficacy in terms of augmenting PS levels on the outer cell membrane (B). Data are shown in means ± SEM. (**C**) After 16 h treatment with 2 μM doxorubicin, HUVECs showed significantly improved ability to activate FX in the presence of tTF-NGR (*p* = 0.0015, **). This effect is due to additional PS on their surface and not to nonspecifc effects following necrosis, since TOPRO3 iodide staining was below 10% and since the increased coagulability could be completely reverted by blocking surface PS by Annexin V binding to PS as shown in the right bars (−doxorubicin, +annexin; +doxorubicin, +annexin, *p* = 0.00003; ***). Data are represented in means ± SEM (one outlier has been excluded from analysis).

**Figure 6:**
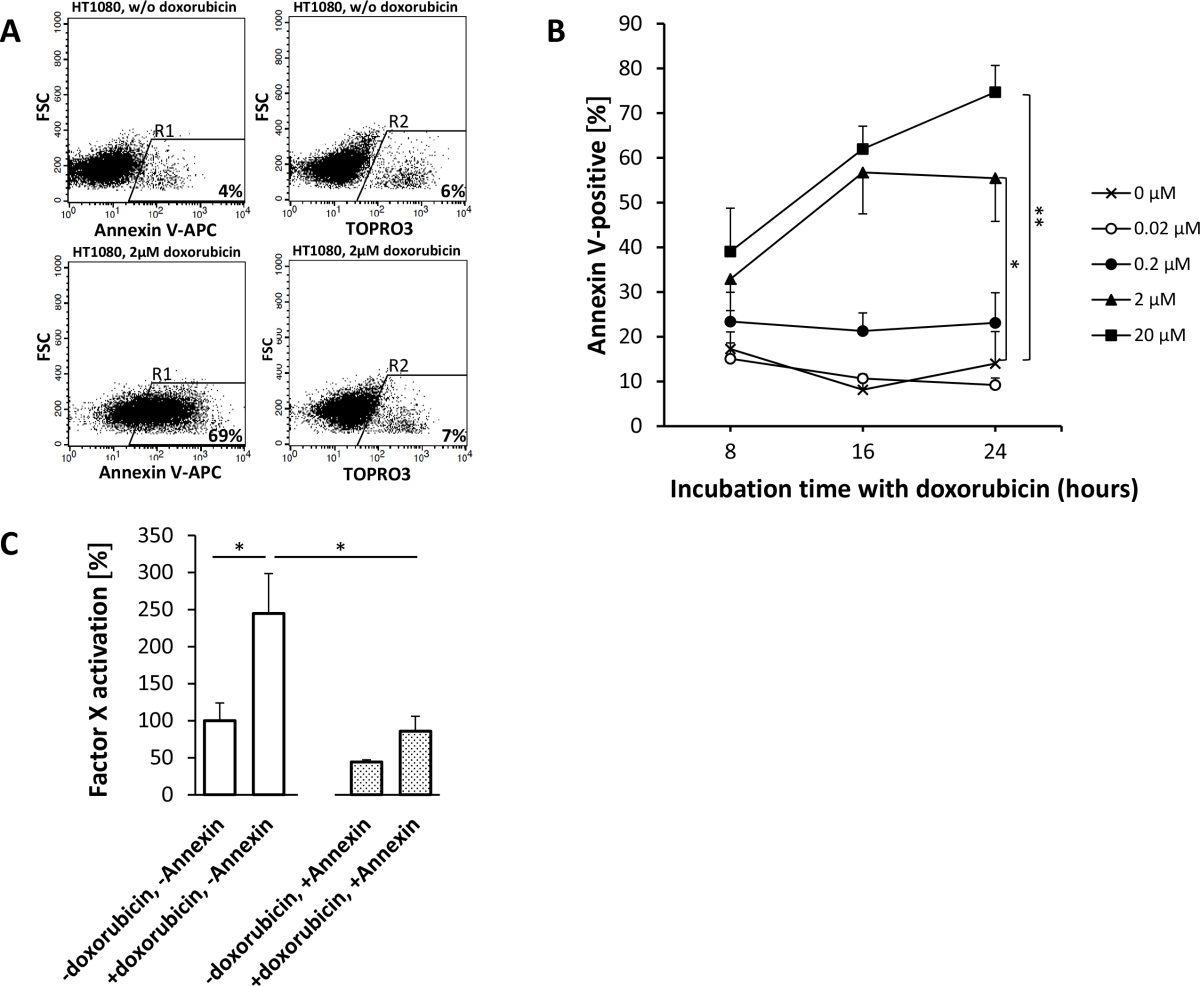
Doxorubicin-induced phosphatidylserine (PS) expression on HT1080 fibrosarcoma cells enhances the procoagulatory activity of tTF-NGR For experimental details see Figure 5. HT1080 fibrosarcoma cells are more susceptible to doxorubicin treatment than HUVECs. Dosages of 0.2 μM doxorubicin yield considerably higher apoptosis levels than controls (0.2 μM: *p* = 0.0559-0.4482; 2 μM: *p*_8h_ = 0.1045, _16h_ = 0.0073, p_24h_ = 0.0260 (*); 20 μM: p_8h_ = 0.0981, p_16h_ = 0.008, p_24h_ = 0.0096 (**); (**A** and **B**). With similar time and dose dependency as in HUVECs PS externalization exceeds HUVECs by up to 100% whereas necrosis (TOPRO3) remained low. If necessary, different gates for APC Annexin V and TOPRO3-stained cells were set to account for distinct staining behaviour. (**C**) As a result, Factor X activation assays show a proportionally increased procoagulatory effect of HT1080 cells upon doxorubicin treatment (*p* = 0.0264, *), which is completely reversible upon PS inhibition by Annexin V (*p* = 0.0268, *). All values are shown in means ± SEM.

**Figure 7:**
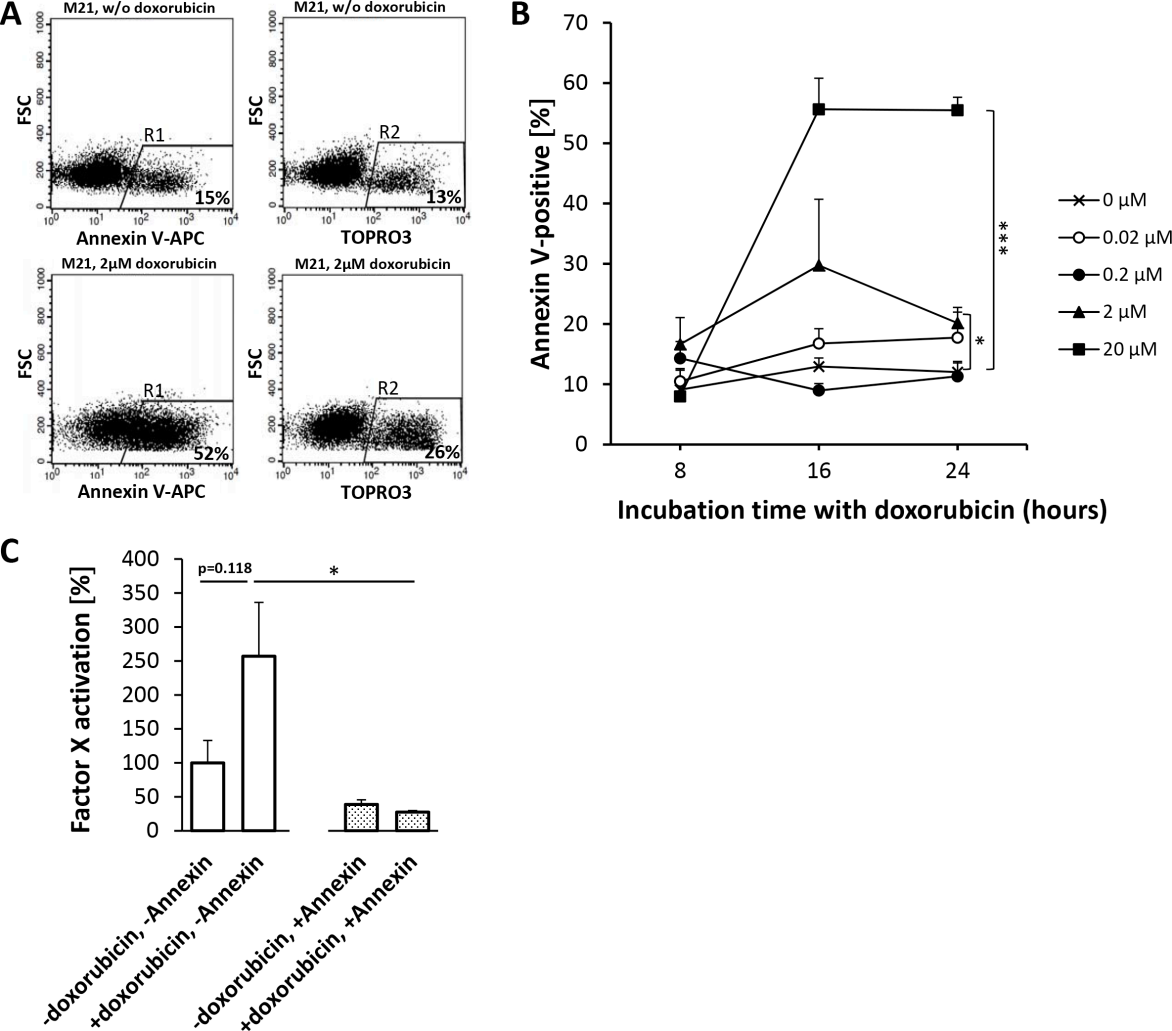
Doxorubicin-induced phosphatidylserine (PS) expression on M21-melanoma cells enhances the procoagulatory activity of tTF-NGR For experimental details see Figure 5. Experiments with M21-melanoma cells showed a clear trend for doxorubicin to enhance PS externalization on the cell surface (**A** and **B**) with a higher degree of simultaneous necrosis than in HUVECs (2 μM: *p*_8h_ = 0.2411, *p*_16h_ = 0.2040, *p*_24h_ = 0.0325 (*); 20 μM: *p*_8h_ = 0.8166, *p*_16h_ = 0.0013, *p*_24h_ = 0.0006 (***)). (**C**) Doxorubicin treatment enhanced the procoagulatory effect of tTF-NGR in the presence of doxorubicin-treated M21 cells, which is entirely reversible upon PS inhibition by Annexin V. Adherent and suspension cells were considered for analysis collectively. Asterisks denote statistical significance (*p* < 0.05, *); all values are presented in means + SEM.

APC Annexin V flow cytometry of HUVEC (Figure 5) and HT1080 (Figure 6) or M21 (Figure 7) tumor cells showed a doxorubicin-induced dose- and time-dependent PS externalization to their outer surface (HUVEC, Figure 5A, 5B; HT1080, Figure 6B; M21, Figure 7B). As shown by FX activation kinetics, HUVEC and HT1080 or M21 tumor cells showed a significantly higher procoagulatory efficacy when used with equimolar concentrations of tTF-NGR upon doxorubicin incubation and higher PS externalization than identical numbers of control cells with low PS externalization (Figures 5C, 6C, and 7C). For proof of a causal relation between doxorubicin-induced PS externalization and procoagulant behaviour of the cells, we could show that preincubation with recombinant human Annexin V, binding and masking PS, completely abolished this elevated procoagulatory state of the cells (HUVEC, Figure 5C; HT1080, Figure 6C; M21, Figure 7C).

### Therapeutic superiority of sequential application of doxorubicin followed by tTF-NGR over both compounds given alone against human tumor xenografts

**Figure 8:**
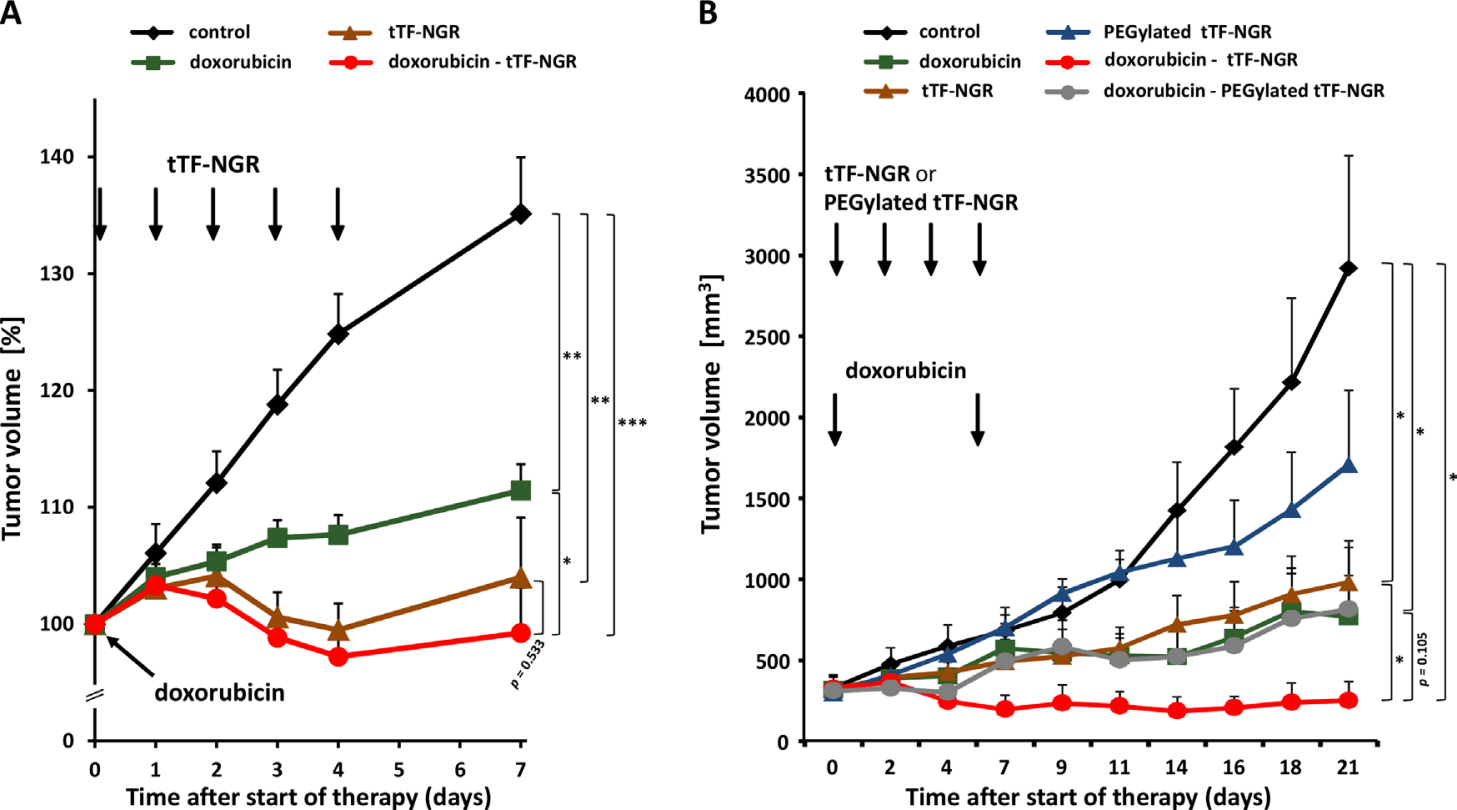
*In vivo* therapeutic activity against tumor xenografts of combinatorial application of doxorubicin - tTF-NGR or - TMS(PEG)_12_ tTF-NGR Drugs were applied intravenously at the doses and time points indicated. Data are presented as means + SEM. Asterisks indicate statistical significance for the groups as indicated (*p* < 0.05, *; *p* < 0.001, **; *p* < 0.0001, ***; Mann-Whitney test). (**A**) Therapeutic activity of doxorubicin (5 mg/kg bw, see arrow; *n* = 13), tTF-NGR at optimal dose (1.5 mg/kg bw, daily application, see 5 arrows; *n* = 16), or the combination of both drugs (*n* = 14) compared to the PBS control (*n* = 15) in a human M21 melanoma xenograft model. Three experiments with CD-1- and BALB/c-nude mice were combined. Start of treatment: 35 days after tumor implantation (CD-1), and 52 days (BALB/c), respectively. Tumor volumes are presented as percentage (start of the therapy = 100%; max. tumor size included into the evaluations was 1.2 cm^3^ at the start of the therapy). In comparison to the PBS control, the therapies with doxorubicin, combination of doxorubicin and tTF-NGR, and tTF-NGR alone revealed highly significant decrease in tumor volumes on day 7. Comparison of the combination therapy versus doxorubicin alone indicates statistical significance (*p* = 0.026), while the comparison with tTF-NGR alone reveals a non-significant trend in favor of the combination (*p* = 0.533). (**B**) Therapeutic activity of doxorubicin (5 mg/kg bw, see arrow; *n* = 5), tTF-NGR (1 mg/kg bw; *n* = 5) or TMS(PEG)_12_ tTF-NGR (5 mg/kg bw; *n* = 4), respectively (both applied every other day, see 4 arrows), or the combination of both drugs (each *n* = 4) at identical doses in a HT1080-human fibrosarcoma xenograft model. In comparison to the PBS control, the therapies with doxorubicin, doxorubicin followed by tTF-NGR, and tTF-NGR alone revealed a significant decrease in tumor volume on day 21 (*p* = 0.0317, *p* = 0.0286, *p* = 0.0259, respectively); comparison of the combination schedule ‘doxorubicin followed by tTF-NGR’ versus doxorubicin alone shows a non-significant trend in favor of the combination (*p* = 0.105), while comparison of ‘doxorubicin followed by tTF-NGR’ versus tTF-NGR alone reveals statistical significance (*p* = 0.0489).

Subsequently, we have tested the combinatorial effect of doxorubicin with subsequent tTF-NGR or random-PEGylated tTF-NGR, respectively, versus saline controls using human M21-melanoma and HT1080-fibrosarcoma xenografts in athymic mice *in vivo.* Drugs and controls (saline) were slowly injected i.v. at the doses and time intervals as shown in Figure 8A and 8B. In Figure 8A, tumor volumes of three human M21 melanoma experiments, including two CD-1 and one BALB/c nude mice trials are presented. Giving tTF-NGR at therapeutically optimal doses (1.5 mg/kg bw, daily × 5) and combining it with 5 mg/kg bw doxorubicin showed better therapeutic efficacy than both drugs given alone and completely stopped tumor xenograft growth for the duration of experimental observation (Figure 8A). In the human sarcoma HT1080 model, tTF-NGR (1 mg/kg bw, 4 times every other day) and random-PEGylated TMS(PEG)_12_ tTF-NGR (5 mg/kg bw, 4 times every other day) were given at suboptimal doses starting 6 hours after the application of 5 mg/kg bw of doxorubicin on day 0 and then repeated on days 2, 4 and 7. In some experiments, this protocol was repeated on days 9 and 11, however without yielding better results (details not shown). With this “days 0, 2, 4, 7” schedule there was significant amplification of the combinatorial therapeutic effect over the tTF-NGR single drug treatment and a clear trend for better therapeutic outcome of the combination when compared with doxorubicin given alone. The combination yielded complete growth suppression of the tumors or tumor remission for prolonged times (Figure 8B). Only a non-significant trend in favour of the combined application of doxorubicin and random-PEGylated TMS(PEG)_12_ tTF-NGR over TMS(PEG)_12_ tTF-NGR alone was detectable and no effect in favour of this combination over doxorubicin alone.

However, in both tumor models reciprocal drug activation leads to an improved therapeutic effect of the combination of doxorubicin and retargeted tTF-NGR over single drugs, completely stopping tumor xenograft growth for the duration of experimental observation, in particular when suboptimal doses and schedules of both tTF-NGR forms were applied.

### Morphology and proliferative capacity of HT1080 tumors upon combinatorial treatment with doxorubicin and tTF-NGR

**Figure 9:**
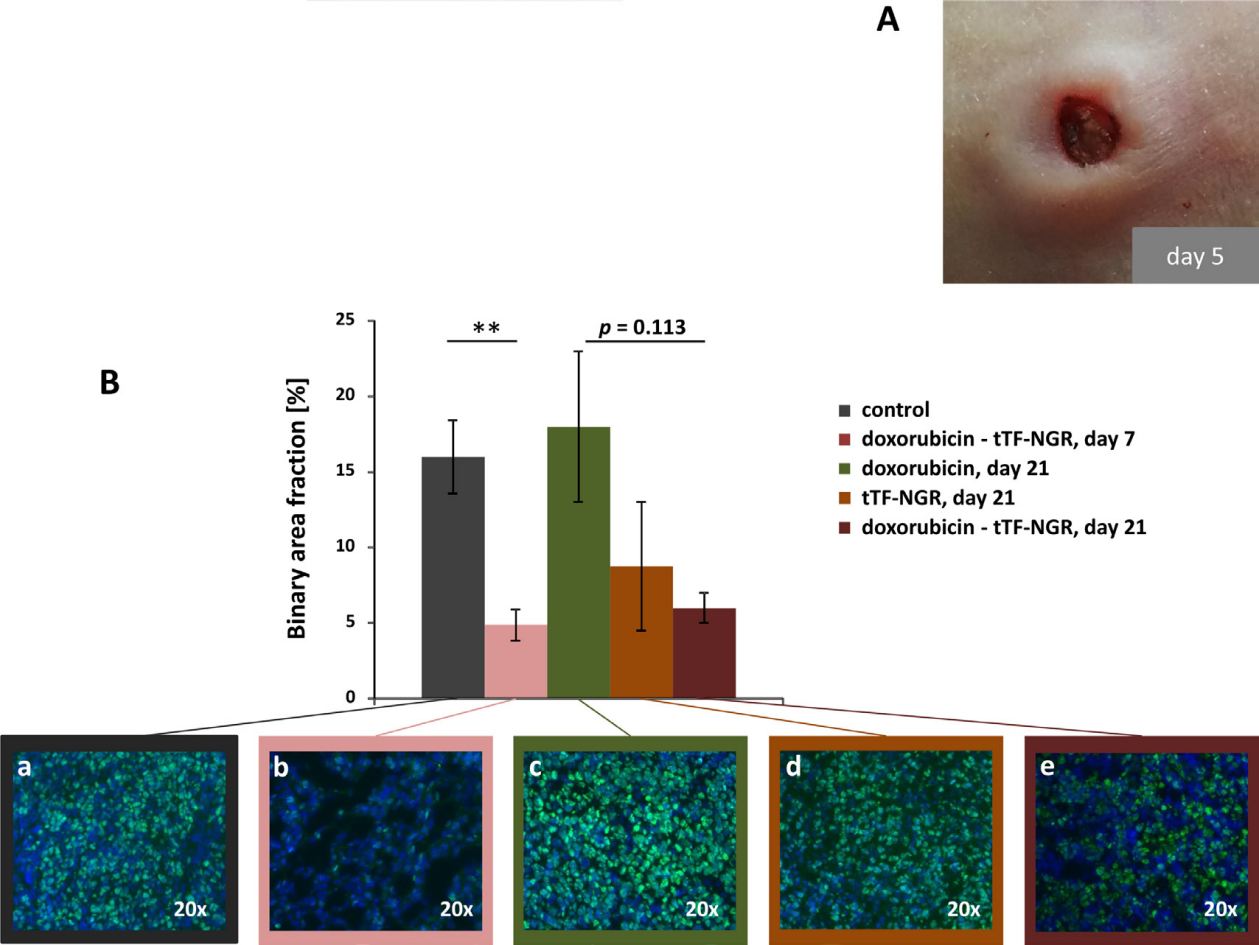
Tumor macroscopic appearance and proliferation as shown by Ki-67 staining (**A**) Appearance of subcutaneous HT1080 tumor after treatment with doxorubicin (5 mg/kg bw, i.v.) followed by tTF-NGR (1 mg/kg bw, i.v.). During the time of treatment (5 days), the tumor collapsed with signs of necrosis. (**B**) Immunofluorescence staining of tumor sections (mean *n* = 2 per group) with anti-Ki67 antibody (green), indicating the proliferation rate in control mice after 21 days (a), in doxorubicin - tTF-NGR treated mice after 7 days (b), in doxorubicin treated mice after 21 days (c), in tTF-NGR treated mice after 21 days (d) and in doxorubicin - tTF-NGR treated mice after 21 days (e). Nuclei were stained with DAPI (blue). The measured binary area fraction (4 sections of each tumor) showed significant decrease of tumor cell proliferative activity in the doxorubicin - tTF-NGR sequence (b) on day 7 over saline control (a; **p = 0.008, Mann-Whitney test). After 21 days proliferative capacity of the doxorubicin monotherapy tumors recovered completely (c), whereas pronounced anti-proliferative effects of the doxorubicin - tTF-NGR sequence (e) over single drugs (c, d) was retained, however, without reaching statistically significant differences comparing the combination vs. doxorubicin (*p* = 0.133) or tTF-NGR (*p* = 0.600) alone.

Macroscopically, the tumors in the combination therapy group showed bluish coloration starting a few hours after injections of tTF-NGR and later central necrotic shrinking as shown in Figure 9A. To test for remaining proliferative activity of the tumor cells following treatment, single tumors were excised in some experiments after treatment and subjected to histological studies. Ki67-stained histology (Figure 9B) showed decrease of tumor cell proliferative activity upon combined therapeutic interventions over saline controls with more pronounced anti-proliferative effects of the doxorubicin - tTF-NGR sequence over single drugs. Moreover, tumors treated with doxorubicin alone, resumed their proliferative capacity 21 days after start of treatment, whereas tumors at this time remained largely non-proliferative upon combination treatment (Figure 9B). As control bars for day 21 (only day 21 shown) are identical with day 7, the combinatorial effect is much deeper than the effect of each single drug even on day 21, but comparable to the combination on day 7, which means that the antiproliferative activity of the combination is prolonged. Interestingly, doxorubicin alone cannot prevent resumed proliferation of the tumors on day 21 at all. This is in contrast to tTF-NGR and in particular to the combination, which inhibits the proliferation of the tumors even on day 21.

These observations again underline the new mechanisms described here for antitumor activity of combinatorial application of a cytotoxic drug followed by induced vascular occlusion via tTF-fusion proteins.

## DISCUSSION

Aminopeptidase N (CD13) is a promising target for delivering therapeutic moieties to tumor vessels and tumor cells. The molecule is increasingly used for experimental *in vivo* imaging of tumors and other angiogenic tissues via different methods [21–32]. Extensive protein expression analysis has revealed a restricted expression of the molecule in normal organs [33].

Our fusion proteins consist of small peptides coupled to the C-terminus of tTF [4–8]. By connecting the peptide to the C-terminus of tTF, the tTF moiety is part of the tTF:VIIa complex adopting a physiological orientation perpendicular to the phospholipid membrane [34], and thus constitutes a potent initiator of coagulation. Peptide-targeted tTF may combine certain advantages over antibodies or larger antibody fragments as targeting moieties for tTF, such as better tumor penetration [35], low non-specific accumulation, low uptake in the reticulohistiocytic system (RHS), and low immunogenicity [36]. The thrombogenic activity of the model compound tTF-NGR in tumor vasculature with subsequent tumor infarction and growth delay could be shown in several tumor mouse models. First-in-man experiences with low doses of tTF-NGR (1–4 mg/m^2^ by i.v. infusion) showed inhibition of tumor perfusion without any side effects as “proof of principle” [5]. NGR-peptide targeting is of further interest, since NGR peptides undergo a non-enzymatic, slow asparagine deamidation to isoaspartate-glycine-arginine (isoDGR) generating a further ligand for alphaV integrins [37, 38], which are also upregulated on tumor endothelial cells [37, 39–41]. Thus, antitumor payloads may be delivered to two target molecules on tumor vasculature in sequence.

Although we could not find synergy of the combination sequence as defined by significant differences between the combination over both single drug in both tumor models, there was combinatorial benefit of the combination with mechanistical specificity for one selected sequence of drugs. Combination effects of targeted tissue factors with other treatment modalities including low-energy ultrasound and liposomal cytotoxics have been observed by us and others before [19, 42]. Doxorubicin represents an organic cytotoxic drug which is widely used in clinical oncology. Here we have shown a completely new mechanism by which tTF-NGR-induced tumor vascular infarction entraps this molecule and potentiates its activity in tumors of different histology. If classical cytotoxic compounds can be captured and entrapped inside tumors so easily and thereby potentiated in their local antitumor activity, this observation possibly bears general importance for the application of cytotoxic drugs in oncology and should be further studied within a wider scope. However, since the liver also showed low, though visible signs of *in situ* apoptosis upon the combination treatment with tTF-NGR and doxorubicin - tTF-NGR combinations, potential liver toxicity should be closely monitored in clinical trials. Interestingly, standard safety pharmacology studies for tTF-NGR according to S6/S9 guideliness of the European Medicines Agency revealed increase of bilirubin as a sign of liver toxicity in dogs.

We have performed the previous series of experiments with the tumor vascular infarction approach mainly in xenograft models of human tumors with different histology [4–8], which provides a large data basis for comparability of the results presented here. tTF-NGR however, is active also in syngeneic metastasis models such as in the B16 melanoma model (data not shown).

No convincing combinatorial effect of the combined application of doxorubicin and the random-PEGylated TMS(PEG)_12_ tTF-NGR was detectable. PEGylation reflects a standard method for attempting improved pharmacodynamic properties of a macromolecule such as the tTF-NGR protein. We are working on a PEGylation program with the objective to improve on the profile of tTF-NGR for clinical application, However, the data presented here show the limitation of this PEGylation approach.

In conclusion, we present a new mechanism by which two different antitumor molecules can reciprocally enhance their respective antitumor activity. In particular, we report tumor entrapment of doxorubicin by targeted tTF-NGR-induced tumor vascular infarction and a better procoagulatory environment for tTF-NGR or random-PEGylated tTF-NGR to yield vascular infarction by doxorubicin-induced apoptosis *in situ*. This observation contributes to the design of randomized clinical trials with tTF-NGR molecules in combination with cytotoxic drugs.

## MATERIALS AND METHODS

### Cloning, expression, purification, and PEGylation of tTF-NGR

Cloning, expression and purification of tTF-NGR was previously described and performed with only minor modifications [4–6]. The expression vector pET-30a(+) (Novagen, Schwalbach am Taunus, Germany), containing the cDNA sequence encoding for the N-terminal HIS_tag_, the tTF-protein (amino acids 1–218 of TF), and the C-terminal heptapeptide GNGRAHA was used. Random TMS(PEG)_12_ PEGylation of the tTF-NGR protein was performed as described before, again with only minor modifications [7]. Briefly, the protein (solved in phosphate buffered saline (PBS)) was incubated for 2 h at 4°C with an 30-fold excess of TMS(PEG)_12_, a trimethyl succinimidyl polyethylene glycol ester (molecular weight: 2420.8 Da), which reacts with primary amino groups (such as lysine) within the tTF-NGR protein, releasing NHS. To remove the NHS group and excess TMS(PEG)_12_, the reaction mixture with the PEGylated protein TMS(PEG)_12_ tTF-NGR was purified by a high performance liquid chromatography (HPLC)-based gel filtration with Sephadex G-25 medium (GE Healthcare, München, Germany). The final PEGylated protein solution was stored at −25°C.

### Cell cultures

Human umbilical vein *endothelial cells* (HUVEC) were purchased from PromoCell (Heidelberg, Germany) in passage 1 and only used at low passage numbers. Cells were cultured in MCDB 131 medium supplemented with 20% fetal calf serum (FCS), 2 mM glutamine (Gibco, Eggenstein, Germany), 50 μg/ml endothelial cell growth supplement (ECGS; Sigma, Taufkirchen, Germany), 5 U/ml heparin (Sigma, Taufkirchen, Germany), and maintained at 37°C in 5% CO_2_ and high humidity. Cell culture dishes were coated with 0.2% gelatine. The human M21-melanoma cell line was kindly provided by Dr. Siletti (University of California, San Diego, CA, USA) and cultured in RPMI 1640 medium supplemented with 10% FCS and 2 mM glutamine. M21 cells grow both adherent and in suspension. The human HT1080-fibrosarcoma cell line was directly obtained from ATCC (Manassas, VA, USA) and cultured in Dulbecco's medium (Gibco) supplemented with 10% FCS. Cell line identity was authenticated and confirmed by short tandem repeat (STR) profiling before and after experiments.

### Phosphatidylserine (PS) staining of endothelial and tumor cells by flow cytometry

Cells were washed once with PBS, trypsinized and harvested. After washing them twice with PBS, cells were resuspended in 100 μl binding buffer according to Schellenberger [43]. To detect early apoptotic cells, one sample was stained with 5 μl APC Annexin V (Becton Dickinson, San Jose, CA, USA) according to the manufacturer's instructions and incubated for 10 minutes in the dark. APC Annexin V binds to cell surface phosphatidylserine which is upregulated on cells undergoing apoptosis. Afterwards, cells were washed twice and finally resuspended in binding buffer. TOPRO3 iodide (Life Technologies, Carlsbad, CA, USA) was added to unstained samples immediately prior to analysis (1 μl, diluted 1:1000) in order to determine the amount of late apoptotic or necrotic cells. On average, 10^4^ cells per sample were counted before apoptosis and viability rates were calculated with BD CellQuest Pro Software (Becton Dickinson). Solely single stains and dyes with excitation by the red diode laser and detection in the FL4 channel (Ex635nm/Em661nm) were used in order to avoid interference with the autofluorescence of doxorubicin. All experiments were conducted at least three times.

### Factor X activation by tTF-NGR in the presence of cells with different amounts of phosphatidylserine (PS) on the surface

The ability of tTF-NGR to enhance the specific proteolytic activation of Factor (F) X by FVIIa was measured by FX activation analysis as basically described by Ruf et al. [44]. In order to assess the differences in the procoagulatory efficacy of tTF-NGR upon binding to HUVECs and tumor cells (HT1080 and M21) with different amounts of PS on their surfaces, we have modified the assay as follows. Briefly, 20 μl of the following was added to each well in a microtiter plate: (a) 50 nM recombinant FVIIa (Novo-Nordisc, Bagsværd, Denmark) in Tris-buffered saline (TBS) containing 0.1% bovine serum albumin (BSA); (b) 750 pM tTF-NGR in TBS-BSA; (c) 25 mM CaCl_2_, and in place of phospholipids (d) 10,000 doxorubicin-treated (2 μM, 16 h incubation) or control cells. To inhibit PS on cell surfaces, cells were incubated with 10 μg/10^5^ cells of recombinant human Annexin V (Becton Dickinson, San Jose, CA, USA) for 15 min before any reagents were added. After 10 min at room temperature, the substrate FX (Enzyme Research Laboratories, Swansea, UK) was added (final concentration 1 μM). After further 10 min, the reaction was stopped in 100 mM EDTA and Spectrozyme FXa (American Diagnostica, Greenwich, USA; final concentration 0.7 mM) was added immediately prior to analysis on a microplate reader (Bio-Rad, München, Germany). The rates of FXa generation were monitored by the development of color at 405 nm, the FX activation by tTF-NGR on cell surfaces was calculated by increase in extinction. Procoagulatory activity within the assay without addition of doxorubicin and/or Annexin V was set as 100%.

### Tumor xenograft models

All procedures on animals were performed in agreement with German regulations (Tierversuchsgesetz §8, Abs. 2) and specifically approved in form of a project license. CD-1 nude mice were purchased from Charles River Laboratories (Sulzfeld, Germany) and acclimated to our animal-experiment facility for at least 1 week before any experimentation. Mice were maintained in individually-ventilated cages (IVC) on a 12:12 h light:dark cycle in a low-stress environment (22°C, 50% humidity, low noise) and given food and water *ad libitum*.

Single tumor cell suspensions were injected subcutaneously (s.c.) into the right anterior flank of female CD-1 or BALB/c nude mice (9–12 weeks old), respectively. For therapeutic experiments, tumor growth was allowed to a mean volume as indicated in the Results section. Mice were randomly assigned to different experimental groups. Doxorubicin, tTF-NGR, randomly PEGylated TMS(PEG)_12_ tTF-NGR, or control 0.9% NaCl solution were slowly applied intravenously (i.v.) via the tail veins in the indicated doses and time schedules. Tumor size was evaluated using a standard caliper measuring tumor length and width; tumor volumes were calculated using the standardized formula (length × width² × π/6). According to our project license, animals had to be sacrificed when tumors became too large, if mice lost too much body weight, or at signs of pain. In this case, mice were sacrificed by cervical dislocation in deep ketamine/xylazine anesthesia in agreement with standard regulations and the project license.

### Fluorescence-based quantification of doxorubicin content in tumor and organ tissues

After final treatment the subcutaneous tumors and organs were excised. Half of each tumor was shock-frozen for fluorescence microscopy; the other half was weighed, homogenized, resuspended in cold PBS containing 1% Triton X-100 (1 ml per 0.1 g tumor tissue) and incubated for 1 h on ice. Afterwards, the suspension was homogenized using ultrasound and incubated with acetonitrile (50% v/v) for 30 min at room temperature. The homogenate was transferred to a 5 ml-centrifugation tube and centrifuged at 3800 rcf for 10 min. The supernatant was taken off and 500 μl of each probe was measured using spectrofluorometric analysis.

Fluorometeric cuvettes are made of quartz glass transparent on all sides. Excitation and emission wavelengths were set according to doxorubicin spectrophotometric properties (Ex470nm/Em580nm). Relative fluorescence signal intensities were visualized in false colors and quantified as arbitrary units (AU). To eliminate nonspecific background fluorescence, data from tissue extracts containing no doxorubicin were subtracted.

### HPLC-based quantification of doxorubicin content in tumor and organ tissues

Doxorubicin content of extracted tissue samples was determined by specific fluorescence and HPLC analysis. A volume of 50 μl of each sample was injected into a Knauer (Herbert Knauer GmbH, Berlin, Germany) gradient reversed-phase (RP)-HPLC system, consisting of two K-1800 pumps, an S-2500 UV detector, a Shimadzu RF10AXL fluorescence detector, and analytical RP-HPLC Nucleosil 100-5 C18 columns (250 mm × 4.6 mm). The parameters were adjusted as follows: solvents (A: water for injection cont. 0.1% TFA, B: acetonitrile, Sigma HPLC-grade cont. 0.1% TFA); flow 1.2 ml per minute; gradient 90% A 0–2 min, 90%–40% A 2–32 min, 40% A 32–42 min, 40%–90% A 42–43 min, 90% 42–45 min. The fluorescence detector was set to 482 nm and 550 nm excitation and emission, respectively, and a high sensitivity with 4× gain. The doxorubicin fluorescence was detected at 22–25 min; the relative signal intensities were quantified as arbitrary units (AU). The recorded data was processed (e.g. integrated) by ChromGate HPLC software (Knauer). To eliminate nonspecific background fluorescence, data from tissue extracts containing no doxorubicin were subtracted.

### Fluorescence-based quantification of apoptosis in tumor and organ tissues *in situ*

Near-infrared fluorescence reflectance imaging (FRI) was performed using the *In Vivo* FX Pro Imaging System (Bruker BioSpin GmbH, Rheinstetten, Germany) equipped with a 400 W halogen illuminator with Cy 7 bandpass excitation (730 nm) and emission filters (790 nm). Fluorescence signals were captured with a cooled charge-coupled device (CCD) camera. Mice received 2 nmol *Annexin vivo* 750 (Perkin Elmer, Waltham, MA, USA) via the tail vein. After 96 h, the animals were sacrificed and the organs were removed and placed on a Petri dish for fluorescence imaging. Fluorescence images were coregistered with the anatomic white light images and quantified using region of interest (ROI) that includes the whole organ. The mean fluorescence intensity of each sample was reported using Bruker MI 7.1 software. Relative fluorescence signal intensities were visualized in false colors and quantified as AU. To eliminate nonspecific background fluorescence, data from tissue extracts containing no doxorubicin were subtracted.

### Histology examination and Ki67 staining

Histological analyses of xenograft tumor tissues were performed with O.C.T.-embedded and cryo-conserved tissues according to standard protocols. Briefly, tissues were embedded in Tissue-TEK O.C.T. (Sakura, Alphen aan den Rijn, The Netherlands), snap-frozen in liquid nitrogen, and stored at −85°C. Frozen samples were cut to 5-μm sections and transferred onto glass slides.

For immunofluorescence staining, slides were dried and sections fixated with acetone according to standard protocols. After three washing steps with PBS, sections were blocked with a 10% bovine serum albumin (BSA, Sigma-Aldrich, Taufkirchen, Germany) solution in PBS for 30 min, again followed by 3 washing steps with PBS. Sections were then incubated with an anti-Ki67 antibody (Abcam, Cambridge, UK; dilution 1:700) overnight at 4°C in a humid chamber in the dark. After another washing step with PBS, the sections were incubated with a fluorescein isothiocyanate (FITC)-labelled goat anti rabbit-IgG antibody (Goat anti-Rabbit IgG (H+L) Secondary Antibody, Alexa Fluor^®^ 488 conjugate, Thermo Fisher, Dreieich, Germany; dilution 1:1000) at room temperature for 90 minutes. Nuclei were stained alongside using 4′,6-diamidino-2-phenylindole (DAPI; Sigma-Aldrich, Taufkirchen, Germany; dilution 1:1000). The sections were mounted with Fluorescence Mounting Medium (DAKO, Hamburg, Germany) and stored at 4°C.

To confirm the Ki67 staining performance, HT1080 cells were used as a positive control. The cells were seeded on 12-mm cover glasses for 4 days (about 85% confluence), followed by two washing steps with PBS. Cells were fixated by incubation with 4% paraformaldehyde (PFA, pH 7.0; stabilized with methanol, Carl Roth, Karlsruhe, Germany) for 10 minutes, followed by 3 washing steps with PBS. The cover glasses were then incubated with 0.2% Triton X-100 (Serva, Heidelberg, Germany; diluted in PBS) for 3 min. Afterwards, the same immunofluorescence staining protocol as for frozen sections (after fixation) has been applied.

5-μm cryo-sections of heart tissue were used as a negative control of the staining performance. BSA (1% in PBS) and normal rabbit IgG (Santa Cruz, Heidelberg, Germany) were used as internal negative controls to prove the specificity of the first and second antibody.

For Ki67, the slides were analyzed using NIS Elements software. Images were acquired in 20× optical zoom for single areas, and in 10× optical zoom for quantitative analysis (binary area fraction) of the whole tumor tissue.

The binary area fraction was calculated by dividing the binary area (absolute content of green fluorescence pixel) above the threshold set by the investigator to measure only specific staining and remaining constant for all sections by the whole tissue area (defined separately by a manual region of interest (ROI) to only asses tissue) presented as percentage.

### Statistical analyses

Statistical significances of differences between the different groups were tested by *t*-test or by Mann-Whitney rank sum test for independent groups. Two-tailed *P* values lower than 0.05 were considered as indicating significant differences. All data are presented in means with SEM.

## Abbreviations

APC: allophycocyanin
APN: aminopeptidase N
AU: arbitrary units
bw: body weight
BSA: bovine serum albumin
C_max_: maximum concentration measured
EDTA: ethylenediaminetetraacetic acid
ICH: International Conference on Harmonisation of technical requirements for registration of pharmaceuticals for human use
IF: immunofluorescence
FRI: fluorescence reflectance imaging
NGR: peptide GNGRAHA
NHS: N-hydroxysuccinimid
PBS: phosphate-buffered saline
PEG: polyethylene glycol
RHS: reticulohistiocytic system
SEM: standard error mean
STR: short tandem repeat
tTF: truncated tissue factor
TMS(PEG)_12_: trimethyl succinimidyl polyethylene glycol ester
RT: room temperature
TBS: Tris-based saline.
